# 晚期非小细胞肺癌抗血管生成药物的联合治疗模式

**DOI:** 10.3779/j.issn.1009-3419.2021.101.16

**Published:** 2021-05-20

**Authors:** 子宜 许, 峻岭 李

**Affiliations:** 100021 北京，国家癌症中心/国家肿瘤临床医学研究中心/中国医学科学院北京协和医学院肿瘤医院 National Cancer Center/National Clinical Research Center for Cancer/Cancer Hospital, Chinese Academy of Medical Sciences and Peking Union Medical College, Beijing 100021, China

**Keywords:** 肺肿瘤, 抗血管生成药物, 靶向治疗, 免疫检查点抑制剂, 联合治疗, Lung neoplasms, Anti-angiogenic agents, Targeted therapy, Immune checkpoint inhibitors, Combination therapy

## Abstract

晚期非小细胞肺癌的治疗方案包括化疗、针对驱动基因的靶向治疗以及以免疫检查点抑制剂为代表的免疫治疗，疗效仍待进一步提高，而联合治疗是当前研究热点。抗血管生成药物在晚期非小细胞肺癌中应用广泛，不仅能通过抑制肿瘤新生血管来抑制肿瘤的生长和转移，还能使血管正常化，调节肿瘤微环境，从而与其他抗肿瘤治疗方式协同增效，共同抑制肿瘤生长。这篇文章通过分析抗血管生成治疗与其他抗肿瘤治疗联合应用的机制，回顾关于大分子单克隆抗体、小分子抗血管生成靶向药物联合其他抗肿瘤方案的临床研究，汇总其疗效及安全性，为晚期非小细胞肺癌的治疗策略提供新的角度和思路。

## Abstract (en)

Treatments for advanced non-small cell lung cancer (NSCLC) include chemotherapy, targeted therapy, and immunotherapy represented by immune checkpoint inhibitors. However, the efficacy of monotherapy is still limited. Nowdays, combination strategy has drawn great attention. Anti-angiogenic agents are widely used in treating advanced NSCLC, which can not only suppress the growth and metastasis of tumor by suppressing tumor vessels, and also have synergic effect with other anti-tumor agents because they can normalize vessels and regulate immune micro-environment. This article summarizes the underlying mechanism of combining anti-angiogenic agents and other anti-tumor agents, reviews the clinical trials on the combination strategy including monoclonal antibodies and tyrosine kinase inhibitor, so as to provide a potential strategy for treating advanced NSCLC.

## 1 晚期非小细胞肺癌治疗现状

肺癌是全球发病率和死亡率最高的恶性肿瘤之一^[1]^。85%的肺癌患者的病理类型为非小细胞肺癌（non-small cell lung cancer, NSCLC），而多数患者初诊时为不可切除的局部晚期或晚期。既往对于晚期NSCLC的标准治疗为细胞毒性化疗药物，但生存获益有限，且不良反应较多，患者耐受性差。免疫检查点抑制剂（immune checkpoint inhibitor, ICI）和针对表皮生长因子受体（epidermal growth factor receptor, *EGFR*）/间变性淋巴瘤激酶（anaplastic lymphoma kinase, *ALK*）等敏感基因突变的小分子靶向治疗药物的出现开启了晚期NSCLC治疗的新时代，彻底改变了晚期NSCLC的治疗策略。在多项大型临床研究的数据基础上，目前国内外指南对于驱动基因突变阳性的晚期NSCLC患者一线推荐使用靶向药物，对于驱动基因阴性且程序性细胞死亡配体（programmed cell death-ligand 1, PD-L1）表达的患者一线推荐使用ICI免疫治疗，相对于传统的化疗，显著延长了晚期患者的无进展生存期（progression-free survival, PFS）和总生存期（overall survival, OS）。然而，靶向药物普遍出现耐药，一代、二代靶向药物耐药时间为9个月-11个月^[2, 3]^，三代靶向药物奥希替尼一线治疗的中位PFS可达到18.9个月^[4]^。免疫治疗也有优势人群的选择，且免疫微环境受影响因素较多，基因通路的改变可能造成免疫超进展，影响免疫疗效。因此，在靶向治疗和免疫治疗基础上的联合治疗，对于改变肿瘤微环境、延缓耐药以及延长患者生存期具有重大意义。

## 2 抗血管生成靶向治疗的基础

肿瘤的发生发展过程中，会在原有血管基础上生成新的血管从而获得血流供给。血管内皮生长因子（vascular endothelia growth factor, VEGF）及VEGF受体（VEGF receptor, VEGFR），尤其是VEGFR-2所介导的信号通路是其中最关键的调节途径，可以调控血管内皮细胞的增殖、迁移、存活以及通透性的改变，在促进血管生成中发挥重要作用。抗血管生成靶向药物通过抑制VEGF与VEGFR的结合，阻断下游通路的激活，可使现有的肿瘤血管系统退化，抑制新血管生成，同时有抗血管通透性，既可抑制肿瘤生长，又提高了化疗的疗效。目前抗血管生成药物主要有以下3类：大分子VEGF-VEGFR单克隆抗体如贝伐珠单抗、雷莫芦单抗，小分子多靶点抑制剂如安罗替尼等，主要针对VEGF、血小板生长因子受体（platelet-derived growth factor receptor, FDGFR）、成纤维细胞生长因子受体（fibroblast growth factor receptor, FGFR）以及重组人血管内皮抑制素。由于目前临床研究证据较多与临床工作应用较广泛的为前两种类型的抗血管靶向药物，因此，本综述将重点探讨前两种类型尤其是贝伐珠单抗和安罗替尼与其他抗肿瘤全身治疗药物的联合治疗模式。

## 3 抗血管生成靶向药物联合治疗模式的前景

早在化疗时代，贝伐珠单抗与化疗的联合就已经得到关注，基于ECOG4599的研究结果，美国食品药品监督管理局（Food and Drug Administration, FDA）在2006年批准将贝伐珠单抗联合含铂双药化疗用于晚期NSCLC的一线治疗，随着BEYOND研究结果更新，2015年该联合方案在我国获批成为一线治疗推荐，延长了晚期使用化疗患者的生存期。随着一代、二代、三代药物的广泛应用，如何延缓酪氨酸激酶抑制剂（tyrosine kinase inhibitor, TKI）药物的耐药成为研究重点。NEJ026研究和JO25567研究结果都证实了在TKI用药初期联合贝伐珠单抗可以延长患者的PFS^[5, 6]^，也为延缓耐药提供了新思路，但在两项研究^[7, 8]^中，联合用药组和TKI单药组之间的OS均无显著差异。CTONG1509的研究^[9]^再次证实TKI联合贝伐珠单抗的PFS获益，2019年国家药品监督管理局（National Medical Products Administration, NMPA）将此联合治疗模式批准用于晚期NSCLC的一线治疗，但是目前其生存期数据尚未成熟，且二代药物与三代药物与贝伐珠单抗联合治疗是否可以取得相似的获益，目前仍缺乏大型研究结果，仍有待进一步探索。

抗血管生成药物与ICI同样可能有协同增效作用，IMPOWER150研究^[10]^证实了ICI与化疗和贝伐珠单抗的四药联合方案显著延长了患者的PFS和OS，小分子抗血管靶向药物与ICI的研究同样取得进展。目前，抗血管生成靶向药物在临床应用广泛，与各种抗肿瘤方案的联合治疗模式可能有协同增效作用，提高了抗肿瘤治疗的有效性。本综述将总结在临床中广泛应用且研究较深入的两种类型抗血管生成药物与不同抗肿瘤治疗的联合模式，从而为肺癌联合治疗策略提供新的思路。

## 4 贝伐珠单抗的联合治疗模式

### 4.1 贝伐珠单抗联合化疗

在化疗时代，驱动基因阴性的晚期非鳞NSCLC一线标准治疗方案为含铂双药化疗，JMDB研究确立了培美曲塞联合铂类作为晚期非鳞NSCLC一线治疗标准方案的地位，该研究结果表明培美曲塞联合顺铂的疗效与吉西他滨联合顺铂的疗效相当，但不良反应发生率更低^[11]^。紫杉醇或紫杉醇联合铂类的方案也是指南推荐的晚期非鳞NSCLC一线治疗之一。贝伐珠单抗是一种重组人单克隆抗体，由于其抗血管生成特性，使肿瘤血管正常化，理论上可以协同化疗药物更好地发挥其抗肿瘤效应。

多项临床研究的阳性结果促使国内外指南推荐贝伐珠单抗作为晚期非鳞NSCLC一线标准治疗。如Ⅲ期临床研究ECOG4599和BEYOND研究^[12, 13]^均发现了紫杉醇和卡铂联合贝伐珠单抗相对单纯化疗可延长患者生存期。一篇*meta*分析纳入4项Ⅱ期、Ⅲ期试验包括AVF-0757、JO19907、ECOG 4599、AVAiL等，共计2, 194例患者，发现在含铂双药化疗方案基础上加用贝伐珠单抗较单用化疗显著延长了患者的中位OS和PFS（死亡：HR=0.90，95%CI：0.81-0.99；病情进展：HR=0.72，95%CI：0.66-0.79）^[14]^。在安全性方面，联合抗血管生成药物治疗使不良反应发生率升高，使用贝伐珠单抗期间发生3级或以上不良事件包括血栓栓塞（8%）、高血压（6%）、出血（4%）、蛋白尿（3%）和肺出血（1%）^[15]^，因此，此联合治疗方案并不适用于易发生空洞、坏死和出血的肺鳞癌，若发生辅助用药无法控制的不良反应也应停药。

在后续维持治疗的研究中，一项Ⅲ期临床研究POINTBREAK^[16]^证实了培美曲塞和卡铂联合贝伐珠单抗后使用培美曲塞联合贝伐珠单抗维持治疗相比紫杉醇和卡铂联合贝伐珠单抗后维持治疗组延长了中位PFS，而另一项Ⅲ期试验AVAPERL研究^[17]^则提示在一线诱导治疗后，使用培美曲塞和贝伐珠单抗维持的患者相比贝伐珠单抗单药维持的患者，中位PFS显著延长，虽然这几项研究结果中不良事件发生率都有不同程度的升高，但是联合治疗并未发现新的安全性问题，不良反应可控。上述两种经典的含铂双药化疗联合贝伐珠单抗的方案除应用于晚期非鳞NSCLC的一线治疗外，还可作为驱动基因阳性患者一线靶向治疗耐药后或驱动基因阴性患者一线免疫治疗进展后的治疗选择。

### 4.2 贝伐珠单抗联合EGFR-TKI

随着分子靶向治疗的研究进展，针对EGFR-TKI的疗效在多项临床研究中得到证实，也因此改变了*EGFR*驱动基因突变的晚期NSCLC患者的治疗策略。虽然EGFR-TKI的广泛应用很大程度上延长了这类患者的生存时间，但获得性耐药的发生不可避免，因此如何延缓耐药和耐药后的机制和策略成为研究热点。临床前研究^[18]^提示在EGFR-TKI用药早期同步联合抗血管生成治疗可持续抑制肿瘤生长，延缓耐药，其作用机制可能与*EGFR*突变影响VEGF通路有关，EGFR的激活可上调VEGF、VEGFR1的表达，促进血管生成，而靶向EGFR可同时阻断VEGF合成，抑制肿瘤血管生成，从而进一步提高抗肿瘤效应^[19]^。

多项临床研究发现一代EGFR-TKI联合贝伐珠单抗疗效良好。一项Ⅲ期临床研究BeTa试验^[20]^对比了厄洛替尼联合贝伐珠单抗和厄洛替尼单药用于晚期NSCLC二线治疗的疗效，其亚组分析发现对于*EGFR*敏感突变的患者，联合治疗的中位PFS比单药治疗更长（17.1个月*vs* 9.7个月），提示联合治疗的价值。在此基础上，日本学者设计了Ⅱ期临床研究JO25567^[21]^对比晚期*EGFR*敏感突变的非鳞NSCLC一线使用厄洛替尼联合贝伐珠单抗和厄洛替尼单药的效果，结果提示联合治疗可明显延长患者的中位PFS（16.0个月*vs* 9.7个月，*P*=0.001, 5）。我国学者开展的Ⅲ期临床研究CTONG1509^[9]^证实了贝伐珠单抗联合厄洛替尼在中国人群中的疗效，联合治疗方案对比厄洛替尼单药治疗的中位PFS显著延长（18.0个月*vs* 11.3个月，*P* < 0.001）。随后另一项Ⅲ期临床研究NEJ026^[5]^发现*EGFR*突变型患者一线使用厄洛替尼和贝伐珠单抗相比厄洛替尼单药可显著延长PFS（16.9个月*vs* 13.3个月，*P*=0.016）。以上研究均提示联合方案为延缓晚期NSCLC患者时间有获益，但JO25567研究后续生存期随访发现联合治疗在OS方面并无明显获益（47个月*vs* 47.4个月，*P*=0.326, 7）^[7]^，而NEJ026结果与其相似，PFS的延长并未转化为OS优势，最终分析发现进展后二线接受奥希替尼治疗患者的PFS以及OS均无明显获益，且3级以上不良反应几乎翻倍^[8]^，而CTONG1509的OS数据尚未成熟。

2020年发表的一篇*meta*分析^[22]^汇总了包括NEJ026和JO25567在内的5项临床研究，结果是一代EGFR-TKI联合贝伐珠单抗对比EGFR-TKI单药可显著延长患者PFS，但对于客观缓解率（objective response rate, ORR）和OS无明显影响，可能与EGFR-TKI单药的反应率较高以及研究随访时间较短有关。安全性方面，联合治疗发生蛋白尿和高血压的不良反应率较单药治疗明显升高。另一篇*meta*分析^[23]^纳入6项研究对比联合治疗和EGFR-TKI或贝伐珠单抗单药治疗的结果，同样发现了PFS方面的显著差异，而在ORR和OS方面未发现显著差异。分析发现联合治疗有更高的1级或2级高血压以及腹泻的发生率。这些研究结果提示，联合治疗的治疗相关不良反应率会随之提高，因此两药的联合应用仍需筛选合适人群。

基于上述PFS获益的研究结果，2020版晚期NSCLC抗血管生成治疗中国专家共识提升了在*EGFR*敏感突变晚期非鳞NSCLC患者中一线应用厄洛替尼联合贝伐珠单抗治疗的推荐等级，并新增了在该人群中应用吉非替尼联合贝伐珠单抗治疗的Ⅱ级推荐。日本一项Ⅱ期临床研究^[24]^纳入42例既往未经系统抗肿瘤治疗的*EGFR*敏感突变晚期NSCLC患者，使用吉非替尼联合贝伐珠单抗作为一线治疗，其ORR为73.8%，中位PFS为14.4个月（95%CI: 10.1-19.2），且*EGFR*外显子19突变的患者中位PFS与*EGFR*外显子21突变的患者有显著差异（18.0个月*vs* 9.4个月，*P*=0.006），提示*EGFR*外显子19敏感突变的患者可能从联合治疗中获益更多。国内一项回顾性临床研究^[25]^对比了一代EGFR-TKI（吉非替尼或厄洛替尼）联合贝伐珠单抗和TKI单药在*EGFR*阳性晚期NSCLC患者一线治疗的真实世界疗效和安全性，结果发现，联合方案取得了比单药治疗更有效的缓解率，ORR分别为95%和74.2%，PFS也显著延长（16.5个月*vs* 12个月，*P*=0.001），3级以上不良反应发生率较单药治疗升高（33% *vs* 14.1%）。

一代EGFR-TKI联合抗血管生成治疗取得了良好效果，而二代及三代EGFR-TKI联合抗血管生成的研究目前没有成熟的数据，但也有一些研究已取得阳性结果。一项Ⅰ期临床研究^[26]^纳入19例*EGFR*敏感突变的晚期NSCLC患者，予以阿法替尼联合贝伐珠单抗作为一线治疗，研究的ORR达81.3%，疾病控制率（disease control rate, DCR）达100%，提示该联合治疗有良好的疾病控制作用。三代靶向药奥希替尼的中位PFS比一代、二代药物更长，但耐药后机制复杂，在研究奥希替尼联合贝伐珠单抗用于晚期*EGFR*敏感突变的NSCLC一线治疗的Ⅰ期或Ⅱ期临床试验中，入组的49例患者有15例（31%）基线有脑转移，12个月PFS率为76%，中位PFS为19个月，ORR为80%，其中6例可评估的脑转移患者ORR达100%^[27]^，提示联合方案有良好的中枢保护作用，疾病缓解率良好，但与既往研究数据相比未增加疗效，仍待大型Ⅲ期临床数据验证联合治疗在晚期NSCLC患者尤其是脑转移患者中的作用。综合各项研究可以发现EGFR-TKI联合抗血管生成治疗具有潜在获益，但仍有需要探讨的问题，比如联合治疗的最佳时机，在治疗开始即同步联用是否比出现缓慢进展时联合疗效更佳；以及最佳适用人群，不同*EGFR*敏感突变类型、共突变类型以及基线有无脑转移的患者群体接受联合治疗的疗效对比，均待更多的大型研究探索。

### 4.3 贝伐珠单抗联合ICI

免疫治疗是近5年发展最快的肺癌治疗方法，以程序性死亡分子-1（programmed cell death-1, PD-1）及其主要配体（PD-1 ligand, PD-L1）抑制剂为代表的ICI改变了NSCLC的治疗现状，但免疫单药治疗的疗效仍有待提高。免疫联合化疗或抗血管生成治疗在多种肿瘤中取得了较单一疗法更优的效果，两种治疗方式可协同增效，延缓肿瘤细胞耐药性的发生^[28, 29]^。免疫治疗和抗血管生成治疗具有多重联合机制。一方面，抗血管治疗可以降低髓源性抑制细胞和调节性T细胞的活性，重塑肿瘤微环境，并且通过阻断VEGF介导的对树突细胞成熟的抑制，使得结合肿瘤抗原的T细胞更有效地启动和活化，增强抗原识别^[30]^；另一方面，抗血管治疗所产生的血管正常化作用可以使大量的T细胞募集到肿瘤部位，实行杀伤作用^[31]^。

IMpower150是第一个探索ICI联合抗血管生成和含铂双药化疗用于晚期非鳞NSCLC一线治疗的疗效和安全性的Ⅲ期研究，已达到研究终点。患者1:1:1随机分配至接受PD-L1抑制剂阿替利珠单抗联合卡铂和紫杉醇（ACP组，402例）、阿替利珠单抗联合贝伐珠单抗、卡铂和紫杉醇（ABCP组，400例）和贝伐珠单抗联合卡铂和紫杉醇（BCP组，400例）。结果显示，ABCP组的PFS显著长于BCP组（中位PFS：8.3个月*vs* 6.8个月；HR=0.62，95%CI：0.52-0.74；*P* < 0.001），中位OS分析显示ABCP组的OS明显长于BCP组（中位OS：19.2个月*vs* 14.7个月；HR=0.78，95%CI：0.64-0.96；*P*=0.02）^[10]^。并且，在肝转移亚组分析中，ABCP组的PFS和OS都比BCP组显著延长，对于预后较差、死亡风险较高的肝转移患者而言是一个新的治疗选择。此外，该研究发现PD-L1表达水平不同的患者均有OS优势，提示获益人群较广，其中PD-L1表达程度越高，患者获益越显著。在不良反应方面，各治疗组免疫相关的不良反应发生率均接近，且未出现新的不良事件，所有不良反应都在预期之内，可控性较高。该研究为晚期非鳞NSCLC患者提供了新的联合治疗模式，OS进一步延长。但目前尚未有研究对比不同类型的ICI联合抗血管生成药物的疗效，且真实世界中的不良反应发生率还待进一步研究。

国内一项真实世界研究^[32]^回顾了69例使用PD-1抑制剂联合抗血管生成药物的晚期NSCLC患者疗效情况，在抗血管生成治疗药物的选择上，有65.2%的患者选择了贝伐珠单抗，整体人群的ORR为31.9%（95%CI: 20.6%-43.2%），中位PFS为8.37个月（95%CI: 6.5-10.0），联合贝伐珠单抗似乎比联合多靶点抗血管靶向药物更有效，PFS有延长趋势（9.0个月*vs* 5.4个月），但并无统计学差异（*P*=0.183）。不良反应发生率为62%，大部分为1级-2级，主要有乏力、食欲下降和恶心等胃肠道反应，仅有2例发生3级不良反应，总体而言该联合治疗方案可耐受。

## 5 小分子抗血管靶向药物的联合治疗模式

盐酸安罗替尼是一种新型小分子多靶点TKI，能有效抑制VEGFR、PDGFR、FGFR、c-Kit等靶点，具有抗肿瘤血管生成和抑制肿瘤生长的作用。ALTER0303研究发现安罗替尼用于晚期NSCLC三线治疗相比于安慰剂组可以显著延长患者的OS（9.63个月*vs* 6.30个月，*P*=0.001, 8），同时，PFS也显著延长达3.97个月（5.37个月*vs* 1.40个月，*P* < 0.000, 1）。与安慰剂对比，安罗替尼治疗的ORR和DCR均得到了显著提高，ORR分别为9.18% *vs* 0.7%（*P* < 0.000, 1）；DCR分别为80.95% *vs* 37.06%（*P* < 0.000, 1），且安罗替尼整体不良反应可耐受^[33]^。

阿帕替尼是一种小分子VEGFR-TKI，可通过抑制VEGFR酪氨酸激酶活性，阻断VEGF与其受体结合后的信号传导，抑制肿瘤血管生成，从而治疗肿瘤。一项Ⅱ期临床研究发现阿帕替尼联合最佳支持治疗对比安慰剂组用于晚期非鳞NSCLC的三线治疗显著延长了患者的PFS（4.7个月*vs* 1.9个月，*P* < 0.000, 1），阿帕替尼组ORR为20.0%，DCR为68.89%。安慰剂组ORR为2.22%，DCR为24.44%，且阿帕替尼耐受良好，常见不良反应为蛋白尿、高血压、手足皮肤反应和恶心呕吐等。3级-4级不良反应多为乏力、高血压、以及手足皮肤反应。相比于静脉用药抗血管靶向药物如贝伐珠单抗、雷莫芦单抗等，安罗替尼和阿帕替尼为口服制剂，更方便肿瘤患者长期服用，患者依从性更高，潜在获益群体更广，成为临床选择之一。

### 5.1 小分子抗血管靶向药物联合ICI

在晚期NSCLC患者的一线治疗中，小分子抗血管靶向药物联合PD-1/PD-L1抑制剂显示出了良好的疗效和耐受性。2019年世界肺癌大会上，我国学者报告了一项关于信迪利单抗联合安罗替尼作为晚期NSCLC一线治疗疗效和安全性的Ⅰb期研究数据，入组患者为驱动基因（*EGFR*/*ALK*/*ROS1*）阴性、未经治疗的Ⅲb期-Ⅳ期NSCLC患者，共纳入22例患者，初步分析结果显示，共计16例患者实现部分缓解，6例患者实现疾病稳定，ORR为72.7%，DCR高达100%^[34]^。该研究在2020年发布了最终分析结果，中位PFS为15个月，1年PFS率为71.4%（95%CI: 47.2%-86.0%），中位OS数据暂不成熟，但1年OS率达95.5%（95%CI: 71.9%-99.3%）。安全性方面，有54.5%的患者发生3级或以上的治疗相关不良反应，但均为可逆毒副反应，仅有1例（4.5%）出现3级以上免疫相关不良反应（免疫相关性肺炎）^[35]^。

在晚期NSCLC患者的后线治疗中，小分子抗血管靶向药物联合PD-1/PD-L1抑制剂同样带来延长复发和OS的希望。2018年美国临床肿瘤学会（American Society of Clinical Oncology, ASCO）国际会议上发布了阿帕替尼联合PD-1抑制剂卡瑞利珠单抗治疗晚期NSCLC患者的Ⅰb期临床研究结果，研究入组27例二线或二线以上化疗失败的晚期NSCLC患者，分别接受卡瑞利珠单抗联合阿帕替尼250 mg或375 mg。结果显示阿帕替尼联合卡瑞利珠单抗的ORR达到41.2%，DCR达到94.1%。阿帕替尼250 mg组的中位PFS达到24周，375 mg组尚在随访中。耐受性考察结果显示卡瑞利珠单抗联合阿帕替尼250 mg组发生1例厌食、1例支气管胸膜瘘，仅1例患者中断卡瑞利珠单抗给药。相对于375 mg组（6例不良反应事件）显示出更好的耐受性及良好的抗肿瘤疗效^[36]^。该研究证实卡瑞利珠单抗联合阿帕替尼治疗NSCLC的有效性及安全性。2019年ASCO国际会议上发布阿帕替尼联合卡瑞利珠单抗治疗晚期NSCLC患者的Ⅱ期临床研究数据，入组96例既往经过一线或一线以上系统化疗的晚期NSCLC患者，ORR为30.8%，PFS为5.9个月^[37]^。

综合疗效和安全性角度，卡瑞利珠单抗联合阿帕替尼250 mg方案显示出更好的耐受性及良好的抗肿瘤疗效，在NSCLC各线治疗中都有望发挥其增强协同效用，为化疗失败的患者提供了新的治疗策略，但对于一线使用免疫治疗的患者群体，即使初始免疫治疗有效（部分或完全缓解），仍有74%的患者会在5年内发生获得性耐药^[38]^，目前针对免疫获得性耐药的治疗策略也在不断探索中。Ⅲ期临床研究OAK试验纳入既往接受一线至二线化疗治疗的局部晚期或转移性NSCLC患者，1:1比例随机分配到PD-L1抑制剂阿替利珠单抗组和多西他赛组，其中阿替利珠单抗组共425例，疾病进展332例，使用阿替利珠单抗疾病进展患者分为3组：继续使用阿替利珠单抗治疗、使用方案规定以外的其他抗癌治疗、不使用其他任何抗癌治疗，结果表明，3组中位OS分别为12.7个月、8.8个月、2.2个月，继续使用PD-L1跨线治疗的患者OS有延长趋势^[39]^。Ⅰb期/Ⅱ期临床研究Keynote 146纳入既往接受过≤二线治疗患者，接受仑伐替尼联合帕博利珠单抗，纳入21例NSCLC患者中既往接受过一线、二线、三线、四线治疗患者数分别为3例、7例、10例、1例，其中既往二线及以上治疗接受过纳武利尤单抗治疗的患者有9例，24周ORR达33%，PFS达5.9个月^[40]^。日本一项回顾性研究^[41]^收集2015年12月-2018年3月使用纳武利尤单抗后使用帕博利珠单抗患者共12例，治疗线数三线、四线分别为4例、8例，中位PFS达3.1个月（1.2个月-12.6个月）。

以上研究结果提示免疫跨线治疗可能为一线免疫治疗后进展的患者进一步延长复发和OS，而此前研究也证实了抗血管生成药物与ICI的协同增效作用，可能使经免疫治疗失败的患者群体获益。目前尚未有在这类群体中应用免疫联合治疗的研究结果公布，但已有多项临床研究正在进行，如Ⅱ期临床研究NCT03689855与NCT03083808、Ⅲ期临床研究NCT04471428与NCT03976375等（表 1）。

**表 1:** 经一线免疫治疗进展后行免疫治疗联合化疗或抗血管生成治疗晚期NSCLC的开展中的临床研究 Ongoing clinical trials on combining immunotherapy and chemotherapy or anti-angiogenic agents in advanced NSCLC patients previously treated with first-line immunotherapy

Study	Phase	Patients	Number	Agents	Primary endpoint
NCT03689855	Ⅱ	NSCLC patients previsously treated with ICI	21	Atezolizumab+rarucirumab	ORR
NCT03083808	Ⅱ	NSCLC patients previsously treated with ICI	35	Pembrolizumab+chemotherapy	DLT; OS
NCT04471428	Ⅲ	NSCLC patients previsously treated with ICI+platinum-based chemotherapy	350	Atezolizumab+kabozantinib vs Docetaxel	OS
NCT03976375	Ⅲ	NSCLC patients previsously treated with ICI+platinum-based chemotherapy	405	Pembrolizumab+lenvatinib vs Docetaxel+lenvatinib	OS; PFS
ORR: objective response rate; DLT: dose limited toxicity; PFS: progression-free survival; OS: overall survival; ICI: immune checkpoint inhibitor; NSCLC: non-small cell lung cancer.					

### 5.2 小分子抗血管靶向药物联合EGFR-TKI

目前，小分子抗血管靶向药物因其有效性、安全性和便利性正在成为科学研究和临床应用关注的热点，而安罗替尼、阿帕替尼与EGFR-TKI联合应用于晚期NSCLC的多项研究正在开展中，目前暂无大型Ⅲ期临床研究的最终结果公布。2020年发表了ACTIVE研究（NCT02824458）^[42]^的初步分析结果，研究旨在探索阿帕替尼联合一代EGFR-TKI吉非替尼一线治疗晚期非鳞NSCLC患者的疗效及安全性，共纳入13例患者，队列A（阿帕替尼500 mg+吉非替尼250 mg组）6例，队列B（阿帕替尼250 mg+吉非替尼250 mg组）7例，共11例患者疗效可评估，ORR为81.8%，DCR为90.9%，总体的中位PFS为13.4个月，队列A的中位PFS为19.2个月，接近RELAY研究的中位PFS数据，而队列B的中位PFS为13.4个月。两个队列的中位PFS无统计学差异，且截至中位随访29.7个月，OS数据暂未成熟。安全性方面，有13例患者观察到不良反应事件，最普遍的不良反应事件为皮疹（84.6%）、腹泻（69.2%）、高血压（69.2%）、蛋白尿（53.8%）和手足综合征（30.8%），均为1级-2级，不良反应均可耐受。研究的初步结果提示了小分子抗血管靶向药物与EGFR-TKI在驱动基因表达阳性的肺癌患者中有应用的前景，但仍需大样本的Ⅲ期临床研究及更成熟的OS数据提供证据支持。

综合以上临床研究的结果，抗血管生成药物与其他抗肿瘤全身治疗的联合应用有潜力成为延缓患者耐药和复发时间、延长患者OS的新的治疗策略，但研究结果仍待丰富，如大型Ⅲ期临床研究的主要终点等更能提供证据，真实世界中运用的效果也是令人期待的数据。总而言之，联合治疗对于晚期NSCLC是新的发展趋势，其中抗血管生成药物联合化疗、靶向治疗以及ICI均可协同增效，不同程度地提高抗肿瘤作用，有助于延缓患者耐药时间，并延长OS，获益群体广，疗效理想，安全性好。
